# Spatial Variation in Abundance Parameters of a Federally Threatened Groundwater Salamander Within and Among Central Texas Headwater Creeks

**DOI:** 10.1002/ece3.71572

**Published:** 2025-06-09

**Authors:** Zachary C. Adcock, Andrew R. MacLaren, Michelle E. Adcock, Michael R. J. Forstner

**Affiliations:** ^1^ Department of Biology Texas State University San Marcos Texas USA; ^2^ Cambrian Environmental Austin Texas USA

**Keywords:** conservation, distribution, groundwater, headwater creek, salamander, threatened species

## Abstract

Semi‐arid conditions in central Texas relegate *Eurycea* salamanders to aquatic habitats influenced by groundwater (i.e., springs, spring‐fed creeks, and subterranean water in alluvium and aquifers). Many studies have noted that epigean (surface) populations of central Texas *Eurycea* occur near spring outlets. Consequently, the U.S. Fish and Wildlife Service designated surface critical habitat units for three species as a uniform distance up and downstream of occupied spring outlets. Here, we used data from visual encounter and quadrat surveys to model relative abundance and relative density, respectively, of federally threatened Jollyville Plateau Salamanders ( *E. tonkawae* ) in relation to downstream distance from a spring outlet in headwater creeks. We additionally use recapture data to investigate movement within these systems and in relation to the critical habitat units. Consistent with other studies, *E. tonkawae* relative abundance and relative density decreased with increasing distance from a spring outlet, and *E. tonkawae* occurred outside of its federally designated surface critical habitat unit at some sites. Importantly, the downstream extent of *E. tonkawae* and the rate of change in relative abundance and relative density varied among sites, which is incongruent with the uniform federal surface critical habitat distance. We observed limited movement within the headwater creeks, with most salamanders recaptured within 5 m of their previous capture location.

## Graphical abstract

Many studies have noted that epigean (surface) populations of central Texas Eurycea salamanders occur near spring outlets, and consequently, the U.S. Fish and Wildlife Service designated surface critical habitat units for three species as a uniform distance up and downstream of occupied spring outlets. Here, we used data from visual encounter and quadrat surveys to model relative abundance and relative density, respectively, of federally threatened Jollyville Plateau Salamanders ( *E. tonkawae* ) in relation to downstream distance from a spring outlet in headwater creeks. Jollyville Plateau Salamander relative abundance and relative density decreased with increasing distance from a spring outlet, but the downstream extent of salamanders and the rate of change in salamander relative abundance and relative density varied among sites, which is incongruent with the uniform federal surface critical habitat distance.

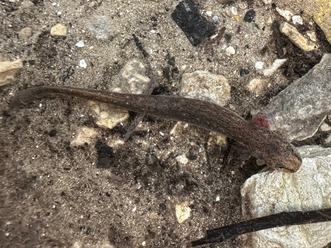

## 1 Introduction

Understanding spatial variation in species distribution and abundance within habitat is a primary objective of ecology and helps inform conservation and management actions (Williams et al. 2002; Mills 2013). Many springs and related groundwater‐dependent ecosystems have fauna adapted to their relatively stable water and environmental conditions, and the distribution and abundances of spring‐associated taxa are often related to the distance from groundwater inputs (Hubbs 1995). Water conditions change and become more variable with distance downstream from a spring due to the influence of ambient air temperature, input from other water sources, runoff, channel morphology, and surrounding habitat. Therefore, the downstream extent of suitable habitat for spring‐associated fauna depends on the interactions of these variables and the volume of groundwater input (Hubbs 1995; Power et al. 1999).

Salamanders are an important faunal component in lotic systems, especially headwaters, because they are often among the top aquatic vertebrate predators, can reach high densities and biomass, and can regulate community structure (Petranka 1998; Davic and Welsh Jr. 2004). The abiotic and biotic factors influencing the distribution and abundance of salamanders in lotic systems have received considerable attention. In eastern North America, salamander distribution in creeks is typically influenced by habitat connectivity, micro‐ and mesohabitat structure, water conditions, and species interactions (e.g., Barr and Babbitt 2002; Lowe and Bolger 2002; Grant et al. 2009; Yeiser and Richter 2015).

Salamanders in the genus *Eurycea* occur throughout the eastern United States and Canada, and they generally demonstrate a biphasic life history and inhabit mesic forests in and near aquatic systems (Petranka 1998). In semi‐arid central Texas, *Eurycea* are fully aquatic and inhabit springs, spring‐fed creeks, and subterranean water in alluvium and aquifers (Sweet 1978, 1982). The evolutionary history of central Texas *Eurycea* was shaped by the inhospitable terrestrial habitat, the relatively stable aquatic conditions provided by spring flows, and the ability of these salamanders to exploit groundwater resources when springs stop flowing (Bruce 1976; Sweet 1977, 1978; Bendik and Gluesenkamp 2013). Many studies have noted that epigean (surface) populations of central Texas *Eurycea* occur near spring outlets (Brown 1942; Bogart 1967; Tupa and Davis 1976; Sweet 1977, 1982; Bowles et al. 2006; Pierce et al. 2010; Gutierrez et al. 2018), and the proximity to a spring is often considered the primary factor that regulates the distribution of salamanders in surface habitat (Sweet 1982; Chippindale and Price 2005). Sweet (1982) suggested that central Texas *Eurycea* are rarely found more than 25 m from a spring outlet because springs provide a reliable source of thermally stable water and usually have minimal siltation and cementation of the substrate.

**FIGURE 1:**
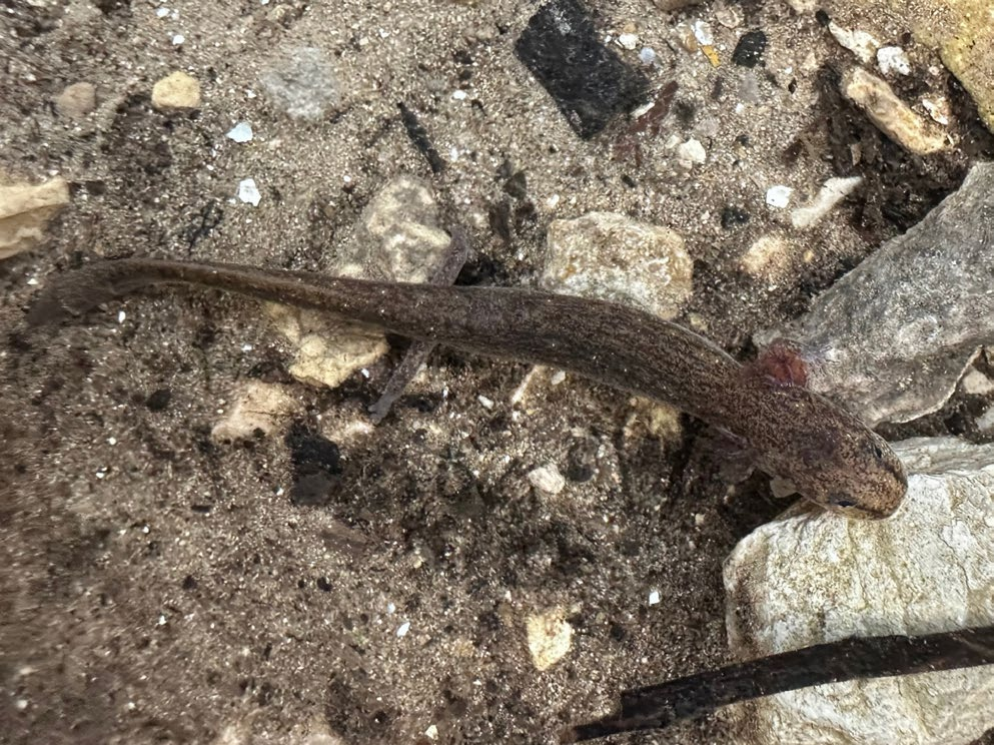
Jollyville Plateau Salamander ( *Eurycea tonkawae* ) in situ at Hill Marsh Spring in Williamson County, Texas, USA.

Jollyville Plateau Salamanders ( *Eurycea tonkawae* ; Figure 1) are one of the approximately 15 species of central Texas *Eurycea*, and they occur in northern Travis and southern Williamson counties (Chippindale et al. 2000; Devitt et al. 2019). In 2013, *E. tonkawae* was listed as threatened by the U.S. Fish and Wildlife Service (2013a). Because it was thought to primarily occur near springs (Chippindale 2005; Bowles et al. 2006), its surface critical habitat was based on the distance to a spring outlet (U.S. Fish and Wildlife Service 2013b). The USFWS designated surface critical habitat units (CHUs) as 80 m up and downstream of known occupied springs, as this represented the furthest distance this species had been observed from a spring outlet at the time of listing (U.S. Fish and Wildlife Service 2013b). However, recent work demonstrates that *E. tonkawae* also use areas further downstream of the CHU distance, gaining creek segments that may not contain discrete spring outlets, and some areas without noticeable groundwater input (Bendik et al. 2016; Adcock, MacLaren, et al. 2020).

The downstream distribution of central Texas *Eurycea* from spring outlets is important to understand for conservation, management, and policy purposes. The primary intent of this study was to estimate how *E. tonkawae* relative abundance and relative density change in relation to distance from a spring outlet in small, headwater creeks. We additionally sought to (1) determine the downstream extent of salamander occurrence within each headwater creek; (2) investigate potential differences in downstream use among size and reproductive classes; and (3) quantify salamander movement within headwater creeks. We discuss these results in the context of the uniform 80 m federal CHU.

## 2 Materials and Methods

### 2.1 Site Descriptions

**FIGURE 2:**
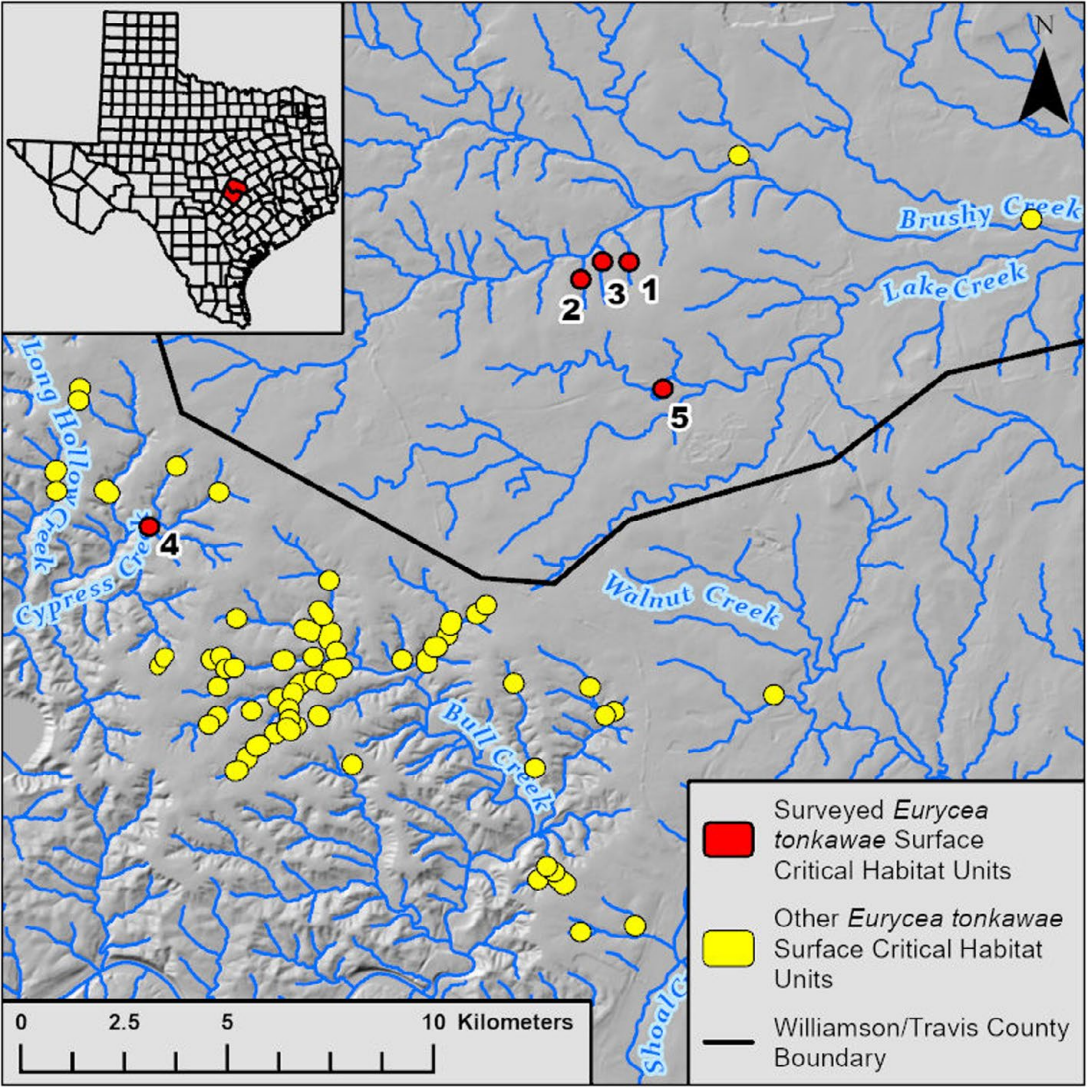
Jollyville Plateau Salamander ( *Eurycea tonkawae* ) survey locations and federally designated surface critical habitat units in Travis and Williamson counties, Texas, USA. Each circle represents the location of a surface critical habitat unit but not the actual size which is an 80 m radius around occupied spring outlets (U.S. Fish and Wildlife Service 2013b). The background is a hillshade layer which is a grayscale, three‐dimensional representation of the terrain surface. This layer has a pixel size (spatial resolution) of 1 m. (1) Avery Deer Spring, (2) Avery Springhouse Spring, (3) Hill Marsh Spring, (4) MacDonald Well, and (5) PC Spring.

We surveyed five spring complexes in Williamson and Travis counties, Texas, USA, that occur within the geographic distribution of *E. tonkawae* (Devitt et al. 2019): Avery Deer, Avery Springhouse, Hill Marsh, and PC Springs in Williamson County and MacDonald Well in Travis County (Figure 2). Each spring complex has a federally designated CHU for *E. tonkawae* (Figure 2; U.S. Fish and Wildlife Service 2013b). Avery Deer and PC Springs both have multiple spring outlets that form two separate spring runs. We treated every spring run as a separate site, for a total of seven sites. We note that the surveyed portions of PC 1 and 2 occur outside of a surface CHU. The USFWS designated critical habitat around another spring within this complex that occurs on private property and that was not accessible for surveys (U.S. Fish and Wildlife Service 2013b). We treated PC 1 and 2 as if they had an 80 m CHU beginning at the spring outlet, as our intention was to compare our results to the federally designated 80 m linear distance.

All seven sites are small, headwater creeks that receive groundwater from the northern segment of the Edwards Aquifer. During periods of heavy rain, some sites may also receive stormwater runoff, but for most of the year, the spring outlet functions as the headwaters of the creek. Here, we refer to a “headwater creek” as a first‐order system where most groundwater discharges from a single location (i.e., spring outlet) as opposed to a “gaining creek” where groundwater enters the system at multiple locations along its length. We discuss the difference between these habitat types because most *E. tonkawae* populations occur in drainages with greater topographic relief that have multiple springs, seeps, and gaining segments along their length (see Figure 2; Davis et al. 2001; Bendik et al. 2016). Although we distinguish between these two system types, we recognize that sites can exist on a continuum between these descriptions. Out of our seven sites, we only noticed downstream groundwater input at Avery Springhouse Spring, at approximately 90 and 110 m downstream, and these seeps were small, inconsistent trickles compared to the spring outlet. The other sites in this study may gain small amounts of groundwater away from the spring outlet, but no visible sources were detected during our thorough surveys. We expect any gaining segments to be intermittent with fluctuating aquifer levels.

The length of the creek surveyed was unequal among the seven sites due to differences in site configuration and access (Table 1). At the Williamson County sites, we surveyed within the entire creek (i.e., the spring outlets to the confluence with another water body). Avery Deer 1 is 68 m long and ends at the start of Avery Deer 2. Avery Deer 2 starts at two spring outlets on opposite banks of the creek and terminates 230 m downstream at a suburban stormwater retention pond. Avery Springhouse confluences with another creek 65.5 m downstream of the spring outlet and eventually ends at a golf course pond 405 m downstream of the spring outlet. Hill Marsh also terminates in a golf course pond but at only 45 m downstream of the spring outlet. PC 1 becomes deep and ponded at 98 m, and PC 2 confluences with PC 1 at 78 m from the spring outlet. At MacDonald Well, we surveyed within a road right‐of‐way, which was a portion of the creek 60–92.5 m downstream of the spring outlet. We justify the inclusion of this site because of the ability to survey within and outside of the *E. tonkawae* federal CHU.

**TABLE 1:** Location information for each Jollyville Plateau Salamander ( *Eurycea tonkawae* ) study site in Travis and Williamson counties, Texas, USA. The number of visual encounter surveys (VES) and quadrat surveys (Quadrats) performed are provided for each site. Surveys were conducted between May 2013 and August 2016.

CHU^a^	Spring complex	Site (Headwater Creek)	Creek length^b^ (m)	VES	Quadrats
6	Avery Deer Spring	Avery Deer 1	68	33	38
6	Avery Deer Spring	Avery Deer 2	230	54	131
6	Avery Springhouse Spring	Avery Springhouse	405	68	203
6	Hill Marsh Spring	Hill Marsh	45	41	102
13	MacDonald Well	MacDonald Well	32.5^c^	16	18
7	PC Spring	PC 1	98	49	110
7	PC Spring	PC 2	78	37	52
*n* = 3	*n* = 5	*n* = 7		*n* = 298	*n* = 654

^a^ Federal critical habitat unit number (U.S. Fish and Wildlife Service 2013b).

^b^ Length of the lotic system. The system ended at the confluence with another creek or a deep, ponded water body.

^c^ The creek length at MacDonald Well refers to the study area (60–92.5 m downstream of the spring outlet), not to the total length of the system.

### 2.2 Salamander Surveys

We surveyed for *E. tonkawae* monthly or every other month from May 2013 to September 2013 and March 2014 to August 2016 using visual encounter surveys (VES) and quadrat surveys. VES are often the most effective technique for rare aquatic amphibians with low densities, and this technique provides a metric of relative abundance (Crump and Scott Jr. 1994; Barr and Babbitt 2001; Vonesh et al. 2010). During VES, each surveyor selected an area(s) to survey based on prior experience and available descriptions of suitable habitat (e.g., Davis et al. 2001; Bowles et al. 2006). Surveyors searched under and in available cover objects for salamanders (Crump and Scott Jr. 1994; Barr and Babbitt 2001; Vonesh et al. 2010). From the onset of the study until February 2015, we always conducted VES within 25 m of a spring outlet, and we also opportunistically conducted VES in areas further downstream. We refer to these as “opportunistic VES” hereafter. Beginning in March 2015, we stratified VES by distance from the spring outlet, and we conducted at least one VES per distance segment per spring complex for the remainder of the study. Distance segments included: (1) 0–25 m, the historically reported area for most central Texas *Eurycea* detections (Sweet 1982; Pierce et al. 2010); (2) 25–80 m, the remaining portion of the *E. tonkawae* surface CHU (U.S. Fish and Wildlife Service 2013b); (3) 80–125 m, outside of the *E. tonkawae* surface CHU (U.S. Fish and Wildlife Service 2013b); and (4) > 125 m, the approximate furthest downstream detection of *E. tonkawae* at these sites during our early survey efforts. We refer to these as “stratified VES” hereafter. For the entire project, we averaged 2.6 VES per spring complex per survey event. The number of VES differed among sites (Table 1) and downstream distance (Table 2) because shorter creeks do not have all of the distance segments, and some of the sites have intermittent flow and are not surveyable during dry conditions (Adcock, Parandhaman, et al. 2020). We measured the upstream and downstream limit of each VES and used the mean distance from the spring outlet for analyses. We surveyed an average of 13.9 linear meters (SD = 12.6, range = 1–55 m) of creek channel per VES. We acknowledge the large standard deviation and range of our survey lengths, but creek configuration substantially influenced the amount of linear creek that was surveyed. For example, a 1 m length of wide creek (i.e., greater than 1 m width) with numerous cover objects can require more survey effort than a 25 m length of narrow creek (i.e., less than 30 cm width) with few, scattered cover objects. Therefore, we also recorded the number of searched cover objects to serve as a measure of effort (Pierce et al. 2010).

**TABLE 2:** The number of visual encounter surveys (VES) and quadrat surveys (Quadrats) performed by distance from a spring outlet. Distances are binned into 20 m segments.

Distance (m)	VES	Quadrats
0–20	162	192
20–40	30	119
40–60	22	66
60–80	12	55
80–100	29	80
100–120	18	32
120–140	10	26
140–160	6	12
> 160	9	72
	*n* = 298	*n* = 654

Although commonly employed, we recognize that the VES methods may not be as rigorous as desired. Therefore, we also conducted quadrats to provide a more controlled and repeatable survey. Quadrats are a labor‐intensive survey method that is useful for determining spatial patterns of aquatic amphibians when densities are high (Jaeger and Inger 1994; Barr and Babbitt 2001; Marsh and Haywood 2010). We surveyed 30 cm × 30 cm quadrats to match the narrow channel width at several of the sites (Adcock et al. 2022). Because quadrats are a known and standard size, the count data provide a metric of relative density (Jaeger and Inger 1994). Quadrats were randomly located using either a systematic or stratified random sampling approach to decrease observer bias of perceived habitat. From the onset of surveys to February 2015, we used a systematic, random sampling approach in which we began at a random point downstream of the spring outlet and performed a quadrat survey every 20 m, on average, moving towards the spring outlet. The random starting position ensured the random placement of the subsequent quadrats (Hayek 1994; Jaeger and Inger 1994). From March 2015 until the conclusion of the study, we used a stratified, random sampling approach (Hayek 1994). Quadrat locations were stratified by distance from the spring outlet using the same segments as VES (i.e., 0–25, 25–80, 80–125, and > 125 m). We sampled three random quadrats per distance segment per spring complex per survey event. The lone exception was at MacDonald Well where we surveyed two random quadrats per distance segment per survey event because the area of access resulted in smaller segments (i.e., 60–80 and 80–92.5 m). For both systematic and stratified sampling schemes, we recorded the distance of the quadrat from the spring outlet to the nearest 0.5 m. We allowed sampling replacement among monthly surveys, but we sampled without replacement on each survey day because quadrats were thoroughly searched and exhausted of all potential cover objects (Jaeger and Inger 1994; Marsh and Haywood 2010). We averaged 6.6 quadrats per spring complex per survey event. The number of quadrats differed among sites (Table 1) and downstream distance (Table 2) because of the random starting position of systematic surveys, shorter creeks do not have all of the distance segments, and some of the sites have intermittent flow and are not surveyable during dry conditions (Adcock, Parandhaman, et al. 2020).

During both VES and quadrat surveys, we moved from downstream to upstream to avoid disturbing areas prior to survey. We recorded the distance from the spring outlet to the nearest 0.5 m for all detected salamanders. We attempted to capture all detected salamanders with small aquarium nets (Sweet 1977; Bowles et al. 2006; Pierce et al. 2010), a sieve, or a Hubbard rake (Adcock et al. 2022). We additionally recorded detection distance and attempted to capture salamanders that were observed but not associated with a VES or quadrat survey (incidental observations). We measured the total length (TL) and snout‐vent length (SVL) of all captured salamanders with dial calipers to the nearest 0.1 mm. We considered all gravid females and salamanders ≥25 mm SVL as adults (Bruce 1976; Sweet 1977; Pierce et al. 2014; Bendik 2017), non‐gravid salamanders between 15 mm and 25 mm as subadult, and salamanders ≤15 mm SVL as juvenile (Bendik and Gluesenkamp 2013). We recorded dorsal photographs of the full body and head of captured salamanders with the animal in a water‐filled container, and we released each salamander at its capture location after processing. We surveyed, captured, and handled all animals in accordance with IACUC 0417_0513_07, Texas Parks and Wildlife Department Scientific Collecting Permit SPR‐0102‐191, and U.S. Fish and Wildlife Federal Permit TE039544‐1.

We sought to identify individual salamander movement within and between headwater creeks. We used Wild‐ID to evaluate the pigmentation patterns on the salamanders' heads to identify potentially recaptured individuals (Bolger et al. 2012; Bendik et al. 2013). Wild‐ID is a pattern extraction and matching program that uses the Scale Invariant Feature Transform (SIFT) algorithm, and the pattern within an image is compared to all combinations of images in a database (Lowe 2004; Bolger et al. 2012). Wild‐ID is vetted as a reliable technique to identify subadult and adult recaptures of *E. tonkawae* (Bendik et al. 2013), and it outperforms several other pattern recognition programs when used for salamanders (e.g., Matthé et al. 2017; Renet et al. 2019). Prior to Wild‐ID evaluation, we cropped photographs to remove as much background as possible, and we standardized the image brightness, contrast, and orientation (Bolger et al. 2012). We compiled the processed images into a single database to potentially document salamander recaptures within and among sites. We manually compared each image to the top 10 potential matches identified by Wild‐ID to determine if an image represented a recaptured or previously “unmarked” individual. We did not rely upon Wild‐ID's match scores because these can be influenced by photograph quality (Bendik et al. 2013). Previous salamander studies report a false rejection rate of less than 5% when the top 10 image matches are reviewed (Renet et al. 2019; Faul et al. 2022). We expect false positives to be negligible because we visually verified each match.

### 2.3 Relative Abundance Analyses

We modeled *E. tonkawae* relative abundance in relation to the distance from the spring outlet using count data from all VES as the response variable. Relative abundance analyses did not include data from quadrat surveys or incidental detections. We fit a series of generalized linear mixed models (GLMMs) using the package ‘glmmTMB’ (Brooks et al. 2017) implemented in R (R Core Team 2024).

Our predictor of interest was the mean distance of each VES from the spring outlet. Additional predictors included the random effect of site, the quadratic effect of day‐of‐year, and sampling method (i.e., opportunistic versus stratified VES). We treated the effects of site as random to not only allow inferences about the seven study sites but also about the larger population of headwater creeks from which the sites are sampled (Kéry 2010; Kéry and Royle 2016; Harrison et al. 2018). Additionally, including site as a random effect accounts for potential differences in abundances among sites, controls for nonindependence of repeated samples within a site, and quantifies the variation among sites (Bolker et al. 2009; Kéry 2010; Kéry and Royle 2016; Harrison et al. 2018). We included the quadratic effect of day‐of‐year to account for known phenological patterns of surface abundance (Kéry and Royle 2016; Edwards and Crone 2021). In surface habitat, the relative abundance of *E. tonkawae* , and closely related congeners, demonstrates an annual peak during spring and summer months and trough in winter months (Bowles et al. 2006; Pierce et al. 2010). This trend corresponds to subadult recruitment and immigration into surface habitat in the summer and adult emigration to subsurface habitat for breeding in the winter (Bendik 2017; Adcock 2022). The quadratic predictor included both the lower‐order term of “day” in addition to “day^2^” to adhere to the rules of marginality (McCullagh and Nelder 1989; Kéry and Royle 2016). We also included sampling method as a binary factor to account for potential differences in abundance between VES conducted opportunistically versus VES stratified by distance segments. Finally, we included a log offset of the number of searched cover objects to account for the known differences in effort among surveys, and we scaled and centered data prior to modeling (Kéry 2010; Kéry and Royle 2016).

We built four models that included a null model, two random intercepts models, and one random intercepts and slopes model. The null model was a model without predictors (i.e., a model of the mean). The random intercepts models allowed for different relative abundances (intercepts) among sites (Zuur et al. 2009; Harrison et al. 2018). These included a model with the random effect of site, the quadratic effect of day‐of‐year, and sampling method, and another model with the same predictors plus mean VES distance as a fixed effect. Data exploration using multivariate plots (Sarkar 2008; Zuur et al. 2009) showed that our study sites may have different patterns in counts per distance. Therefore, we also included a random intercepts and slopes model that allowed for different relative abundances (intercepts) and for different relationships between counts and mean survey distance (slopes) among sites (Zuur et al. 2009; Harrison et al. 2018). Site and mean VES distance were included as random effects and the quadratic effect of day‐of‐year and sampling method as fixed effects (Table 3).

**TABLE 3:** Model selection results assessing relative abundance and relative density of Jollyville Plateau Salamanders ( *Eurycea tonkawae* ) in relation to distance from a spring outlet. The model parameters, degrees of freedom (df), Akaike's Information Criterion corrected for small samples (AIC_*c*_), difference in AIC_*c*_ from the top model (∆ AIC_*c*_), model weight (ω_i_), marginal *R* ^*2*^, and conditional *R* ^*2*^ are provided for each model. Model parameters include headwater creek (site), distance from the spring outlet (distance), the quadratic effect of day‐of‐year (day + day^2^), and sampling methodology (method). Site and distance are identified as a random effect (^RE^) or fixed effect (^FE^) per model.

Model	df	AIC_*c*_	∆ AIC_*c*_	ω_i_	Marginal *R* ^*2*^	Conditional *R* ^*2*^
**Relative abundance**						
Site^RE^ + distance^RE^ + day + day^2^ + method	8	1170.3	0.0	> 0.999	0.528	0.825
Site^RE^ + distance^FE^ + day + day^2^ + method	7	1186.1	15.7	< 0.001	0.477	0.618
Null	2	1358.7	188.4	< 0.001	—	—
Site^RE^ + day + day^2^ + method	6	1371.5	201.2	< 0.001	0.108	0.171
**Relative density**						
Site^RE^ + distance^RE^ + day + day^2^ + method	8	692.1	0.0	0.830	0.733	0.962
Site^RE^ + distance^FE^ + day + day^2^ + method	7	695.3	3.2	0.170	0.839	0.895
Site^RE^ + day + day^2^ + method	6	826.4	134.2	< 0.001	0.112	0.216
Null	2	848.7	156.6	< 0.001	—	—

We first determined the best fit error distribution. We initially fit the global model to a Poisson distribution because our response variable was *E. tonkawae* counts (Kéry and Royle 2016; Brooks et al. 2017). The ratio of residual deviance to the residual degrees of freedom indicated overdispersion, so we fit the global model to a type II negative binomial (NB) distribution (Kéry and Royle 2016). Many VES had zero counts. To test for zero‐inflation, we also fit the global model to a zero‐inflated Poisson (ZIP) and zero‐inflated type II negative binomial (ZIB) error distributions (Brooks et al. 2017). We then compared the global model with Poisson, NB, ZIP, and ZIB distributions with Akaike Information Criterion corrected for small samples, AIC_*c*_ (Burnham and Anderson 2002) and determined a NB was the best fit error distribution for further analyses (Brooks et al. 2017).

We fit the null, two random intercepts, and one random intercepts and slopes models using the NB error distribution and compared the four models using an information theoretic approach (Burnham and Anderson 2002). We calculated ∆ AIC_*c*_, the difference between the AIC_*c*_ value of a particular model and the lowest AIC_*c*_ value of all the models. We considered the model(s) with the lowest AIC_*c*_ score to be the top model(s) and we considered models with ∆ AIC_*c*_ ≤2 to be competing models with support for making inference (Burnham and Anderson 2002). We determined the importance of covariates with a Wald z‐test (Bolker et al. 2009). We assessed model fit with marginal and conditional *R* ^*2*^ according to Nakagawa and Schielzeth (2013) and Johnson (2014), and model adequacy by evaluating plots of residuals versus fitted values and explanatory values as well as a qqplot of random effect means (Harrison et al. 2018).

To visually compare intercepts and slopes among sites, we predicted counts with a 95% confidence interval (CI) for the top model. We held day‐of‐year constant at the mean survey day (day 178), sampling method constant at “stratified VES”, and the effort offset constant at 100 searched cover objects for predictions. We predicted counts and CIs for the entire downstream distances of Avery Deer 1, Hill Marsh, PC 1, and PC 2. We truncated predictions to the distances for which we surveyed at MacDonald Well, and to the upper 150 m of Avery Deer 2 and Avery Springhouse.

### 2.4 Relative Density Analyses

We also modeled *E. tonkawae* relative density compared to the distance from the spring outlet using count data from all quadrat surveys as the response variable. Relative density analyses did not include data from VES or incidental observations. We used the same modeling procedure, response variable (count), random effect of site, and quadratic effect of day‐of‐year as described for the relative abundance models. We again included sampling method as a binary factor to account for potential differences in salamander density between quadrats randomized by systematic or stratified methods. The predictor of interest was the distance of the quadrat from the spring outlet to the nearest 0.5 m. We built the same four models as in the relative abundance analyses (Table 3). However, we did not include an effort offset because quadrats were identical in size, and the counts per area provide an index of salamander density. The NB distribution was also the best fit error distribution for quadrat data. We used the same methods to compare and assess models and predict counts. The mean survey day used for predictions was day 180, and we held sampling method constant as “stratified”.

### 2.5 Counts as Indices

We lacked adequate salamander recaptures to analyze VES and quadrat data in a capture‐mark‐recapture framework (see 3. Results), and these populations were open to demographic changes between survey events. Consequently, we used counts as an index to model relative abundance for VES data and relative density for quadrat data. It is important to note that counts confound absolute abundance and density with detection probability (Williams et al. 2002; Kéry 2010). Indices yield weaker inferences, and detection‐naïve estimates of abundance perform poorly if detection error is present (Williams et al. 2002; Dénes et al. 2015; Kéry and Royle 2021). Comparing relative abundance and density among factors and covariates requires that we assume detection probability is constant, on average, over the variables of interest over the dimension of comparison (Hyde and Simons 2001; Williams et al. 2002; Kéry 2010; Kéry and Royle 2021), and this assumption is commonly violated for amphibians (Hyde and Simons 2001; Bailey et al. 2004; Dodd Jr. and Dorazio 2004; Mazerolle et al. 2007). While detection probability likely contributes to variability in our data, it is unlikely to explain all the variation, and we assume that any trend in detection probability associated with distance from the spring outlet is small in magnitude compared to trends in actual abundance and density (Kéry and Royle 2021). We chose to analyze these data with GLMMs as opposed to N‐mixture models (Royle 2004) because we lacked demographic closure between monthly surveys. Further, regression of count data yields reliable inference on relative abundance and comparable results to N‐mixture models (Barker et al. 2018).

## 3 Results

**FIGURE 3:**
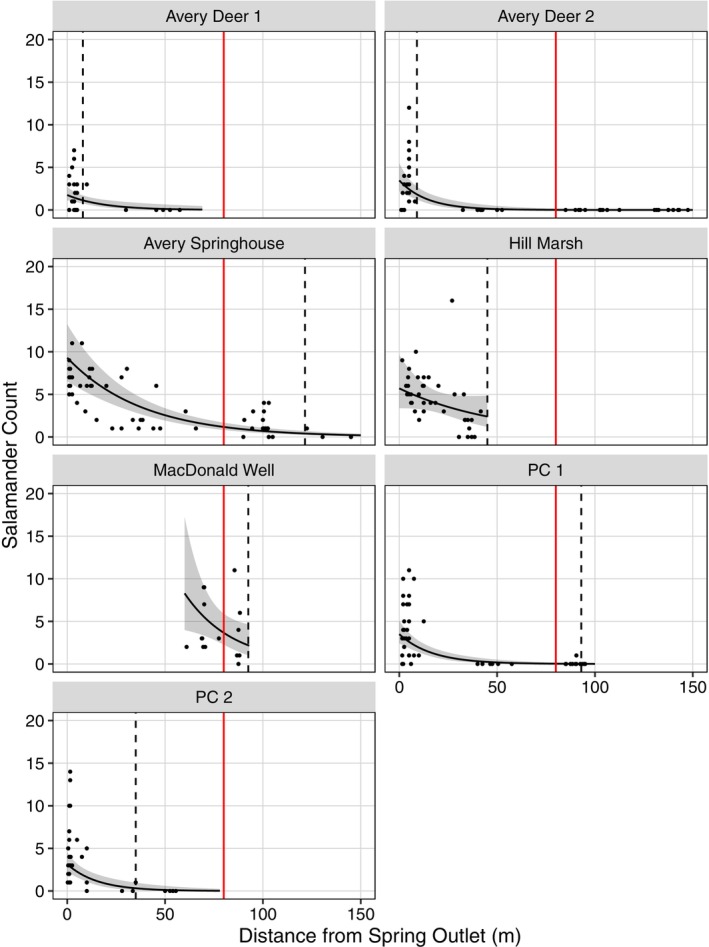
Relative abundance of Jollyville Plateau Salamanders ( *Eurycea tonkawae* ) compared to distance from the nearest spring outlet in seven headwater creeks in Travis and Williamson counties, Texas, USA. Counts (lines) and 95% confidence intervals (dark shaded regions) are predictions from the top generalized linear mixed model with day‐of‐year held constant at the mean survey day (Day 178), sampling method held constant at “stratified VES”, and a log offset of effort held constant at 100 searched cover objects. Raw salamander counts (points) are not standardized by the number of searched cover objects, and one point at PC 2 exceeds the y‐axis limit. The vertical red line represents the limit of the federally designated critical habitat unit, i.e., 80 m from the spring outlet (U.S. Fish and Wildlife Service 2013b). The vertical dashed line denotes the furthest downstream detection of *E. tonkawae* in each headwater creek.

**FIGURE 4:**
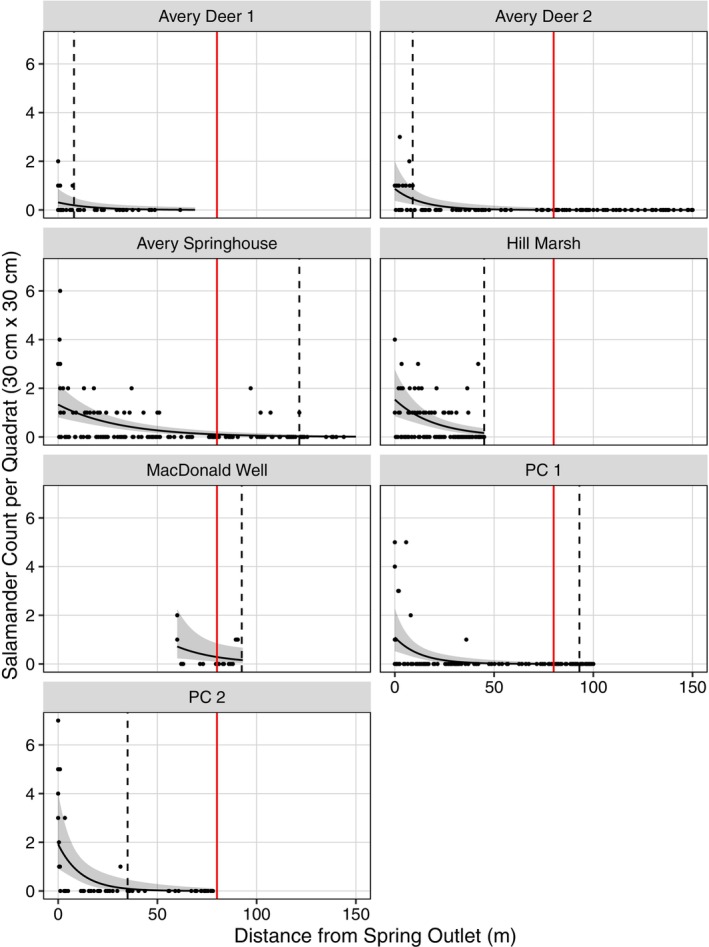
Relative density (counts per 30 cm × 30 cm quadrat) of Jollyville Plateau Salamanders ( *Eurycea tonkawae* ) compared to distance from the nearest spring outlet in seven headwater creeks in Travis and Williamson counties, Texas, USA. Predicted counts (lines) and 95% confidence intervals (dark shaded regions) are from the top generalized linear mixed model with day‐of‐year held constant at the mean survey day (Day 180) and sampling method held constant at “stratified”. Points are raw salamander counts. The vertical red line represents the limit of the federally designated critical habitat unit, i.e., 80 m from the spring outlet (U.S. Fish and Wildlife Service 2013b). The vertical dashed line denotes the furthest downstream detection of *E. tonkawae* in each headwater creek.

We conducted 298 VES and 654 quadrats among the seven sites (Tables 1 and 2). These included 88 opportunistic VES, 210 stratified VES, 259 systematic quadrats, and 395 stratified quadrats. We detected a total of 1216 *E. tonkawae* and captured 806 in this study: 934 detections and 594 captures in VES, 190 detections and 142 captures in quadrats, and 92 detections and 70 captures from incidental observations. The furthest detection downstream of the spring outlet at each site was: 8 m at Avery Deer 1, 9 m at Avery Deer 2, 121.5 m at Avery Springhouse, 45 m at Hill Marsh, 92.5 m at MacDonald Well, 93 m at PC 1, and 35 m at PC 2 (Figures 3 and 4). We frequently detected salamanders throughout the surveyed portion of MacDonald Well, the entire 45 m creek at Hill Marsh, and the upper 120 m of Avery Springhouse. In contrast, only six total *E. tonkawae* were detected beyond 10 m from a spring outlet at the other four sites: none at Avery Deer 1 or 2, four at PC 1 (two at 15.5 m and one each at 36 and 93 m), and two at PC 2 (31.5 m and 35 m). For comparison, we detected 563 salamanders at these four sites: 58 at Avery Deer 1, 103 at Avery Deer 2, 198 at PC 1, and 204 at PC 2.

We detected a total of 67 *E. tonkawae* >80 m from a spring outlet (i.e., outside of the federally designated CHU distance): 40 at Avery Springhouse, 26 at MacDonald Well, and one at PC 1. Respectively, these constituted 13.3%, 32.9%, and 0.5% of observations at these sites. The most downstream observations at Hill Marsh and MacDonald Well were at the survey limits.

### 3.1 Relative Abundance Modeling

The random intercepts and random slopes model was the top model (ω_i_ > 0.999) with no competing models within two AIC_*c*_ units (Table 3). The fixed effects (marginal *R* ^*2*^) explained 0.528 of the variance, and the combination of fixed and random effects (conditional *R* ^*2*^) explained 0.825 of the variance (Table 3). Mean distance of VES (β = −2.882, *p* < 0.001) and the quadratic effect of day‐of‐year (β = −0.182, *p* = 0.002) were significant predictors of relative abundance (Table 4). Salamander relative abundance was significantly lower in stratified VES compared to opportunistic VES (β = −0.752, *p* < 0.001; Table 4).

**TABLE 4:** Summary of the model parameters assessing Jollyville Plateau Salamander ( *Eurycea tonkawae* ) relative abundance and relative density in relation to distance from a spring outlet. Model parameters include headwater creek (site), distance from the spring outlet (distance), and the quadratic effect of day‐of‐year (day + day^2^), and sampling methodology (method). Random effects are identified as “^RE^”.

Parameter	Overdispersion parameter (σ)	Random effect variance	Estimate (β)	Standard error	*Z*‐value	*p*
**Relative abundance**	2.18					
Site^RE^		τ_00_ = 3.276				
Distance^RE^		τ_11_ = 1.845				
Intercept			−4.127	0.741	−5.567	< 0.001
Day			−0.123	0.062	−2.003	0.045
Day^2^			−0.182	0.058	−3.114	0.002
Distance			−2.882	0.682	−4.228	< 0.001
Method (stratified)			−0.752	0.128	−5.883	< 0.001
**Relative density**	1.24					
Site^RE^		τ_00_ = 4.194				
Distance^RE^		τ_11_ = 3.850				
Intercept			−4.103	1.111	−3.694	< 0.001
Day			−0.089	0.110	−0.814	0.416
Day^2^			−0.366	0.105	−3.491	< 0.001
Distance			−5.075	1.288	−3.942	< 0.001
Method (systematic)			0.269	0.209	1.292	0.196

The random intercept variance (τ_00_) was 3.276 and indicates the variability in counts among sites. Avery Springhouse and Hill Marsh had the highest predicted relative abundance at the spring outlet, and MacDonald Well and Avery Springhouse had the highest predicted relative abundance at the CHU boundary when sampling method was held constant as stratified VES (Figure 3). The random slopes variance (τ_11_) was 1.845 and indicates the variability in counts per mean distance. Although slopes varied among sites, all were negative indicating relative abundance decreased with increasing distance from the spring outlet at all sites (Figure 3).

### 3.2 Relative Density Modeling

The random intercepts and random slopes model was the top model (ω_i_ = 0.830) with no competing models within two AIC_*c*_ units (Table 3). The fixed effects (marginal *R* ^*2*^) explained 0.733 of the variance, and the combination of fixed and random effects (conditional *R* ^*2*^) explained 0.962 of the variance (Table 3). Distance of quadrat (β = −5.075, *p* < 0.001) and the quadratic effect of day‐of‐year (β = −0.366, *p* < 0.001) were significant predictors of relative density (Table 4). Salamander relative density was not different in systematic quadrats compared to stratified quadrats (β = 0.269, *p =* 0.196).

The random intercepts variance (τ_00_) was 4.194 and indicates the variability in counts among sites. Avery Deer 1 and 2 had the lowest predicted relative density at the spring outlet, and MacDonald Well and Avery Springhouse had the highest predicted relative density at the CHU boundary when sampling method was held constant as stratified quadrats (Figure 4). The random slopes variance (τ_11_) was 3.850 and indicates the variability in counts per distance. Although slopes varied among sites, all were negative which indicates relative density decreased with increasing distance from the spring outlet at all sites (Figure 4).

### 3.3 Size Class and Reproductive Status

Of the 806 captures, 542 were adults, 210 were subadults, and 54 were juveniles. Salamander classes demonstrated similar mean capture distances from the spring outlet: adults ($\bar{x}$ = 17.08, SD = 28.77 m), subadults ($\bar{x}$ = 19.74, SD = 29.91 m), and juveniles ($\bar{x}$ = 21.54, SD = 21.56 m). Adult counts included 44 gravid females. Gravid ($\bar{x}$ = 23.64, SD = 29.48 m) and non‐gravid ($\bar{x}$ = 17.75, SD = 28.60 m) salamanders also demonstrated similar mean capture distances from the spring outlet.

### 3.4 Movement

We recorded dorsal head photographs for 789 of the 806 captures, and using Wild‐ID, we identified 75 image matches (i.e., recaptured salamanders). These 75 recaptures were of 61 individuals (11 individuals were recaptured more than once). We recorded distance data for 73 of the 75 recapture events. Our treatment of individual spring runs as separate sites was justified by our capture‐mark‐recapture results. We did not document a salamander moving between any of the headwater creeks, including no movement between Avery Deer 1 and 2 or PC 1 and 2 where the creeks are connected.

The mean distance moved between capture occasions was 2.3 m (SD = 4.7 m), and the mean time between captures was 116.8 days (SD = 100.5 days). The mean rate of movement was 0.0472 m/day (SD = 0.1668 m/day). We recaptured 66% (*n* = 48) of *E. tonkawae* ≤1 m from its previous capture location, and we recaptured 11% (*n* = 8) of *E. tonkawae* ≥5 m from its previous capture location. Seven of the eight movements ≥5 m in distance were in the upstream direction. The maximum observed movement was 30 m over 22 days (1.364 m/day) at Avery Springhouse which was an upstream movement from 118 to 88 m. The second furthest movement was 19.5 m over 91 days (0.214 m/day), and was also an upstream movement at Avery Springhouse from 20 to 0.5 m. We did not recapture enough salamanders (*n* = 7) outside of the CHU distance to formally compare movement patterns within and outside of the CHU. However, we note that three of the eight (37.5%) movements ≥5 m in distance occurred outside of the CHU including the maximum observed movement event.

## 4 Discussion

### 4.1 Relative Abundance and Density

The relative abundance and density of *E. tonkawae* were highest at the spring outlet and decreased with distance away from the spring (Figures 3 and 4). This pattern was observed at all sites, even though *E. tonkawae* relative abundance and density were different among sites (Table 4 and Figures 3 and 4). This supports previous results for this species (Bowles et al. 2006; Bendik et al. 2014, 2016) and other central Texas *Eurycea* salamanders (Tupa and Davis 1976; Sweet 1982; Pierce et al. 2010). While the general decreasing pattern was observed at all sites, the rate (slope) at which relative abundance and density decreased was different among sites (Table 4 and Figures 3 and 4). It is not surprising that downstream patterns of relative abundance and density would differ among sites, but the documentation of this variation is novel for these taxa. Further, this finding is important because the federally designated CHUs are a uniform distance.

Sampling method was significant for relative abundance estimates (i.e., opportunistic versus stratified VES) but was not for relative density estimates (i.e., systematic versus stratified quadrats). This is not surprising as the implementation of a stratified design removed a layer of surveyor bias for VES. In contrast, quadrat locations were randomized throughout the entire study, albeit using different randomization techniques. Sampling method was held constant as “stratified” for both relative abundance and relative density model predictions to compare intercepts and slopes. The slope of relative abundance was similar to the slope for relative density at each site. Further, salamander relative abundance and relative density at the CHU boundary were estimated to be highest at MacDonald Well and second highest at Avery Springhouse. Therefore, we observed generally congruent results among the two parameter estimates (Figures 3 and 4) irrespective of the difference in VES and quadrat survey techniques. Our relative abundance and density results do not incorporate an estimate of detection probability. However, we believe the strong negative trend in these estimated parameters reflects real differences, and detection patterns are unlikely to account for all of the observed variation.

Our sampling was limited to portions of the creek 60–92.5 m downstream of the spring outlet at MacDonald Well. It is unclear if the same decreasing trend would be observed if we had samples from up and downstream of the surveyed segment. Bendik et al. (2014) reported a mean density of 1.87 salamanders/m^2^ in a 61.9 m^2^ survey area near the spring outlet of MacDonald Well, but our methods and estimates are different from theirs and not easily comparable.

Congruent with downstream patterns of relative abundance and density, the furthest downstream salamander detections differed among sites. Salamander detections were common beyond the surface CHU distance at Avery Springhouse and MacDonald Well. In contrast, salamanders were rarely detected beyond 10 m of the spring outlet at Avery Deer 1 and 2 and PC 1 and 2, although all have creek sections beyond 10 m that appear suitable for central Texas *Eurycea* salamanders. It is unclear if the rare downstream detections at PC were due to active movement (e.g., dispersal, foraging) or passive drift. The four detections at PC 1 occurred after heavy rainfall events in April 2015 and March 2016. It is possible that these salamanders were flushed downstream by flood waters, but surveys at PC 1 and our other study sites did not yield downstream observations after similar large storm events (e.g., October 2013, May 2015, October 2015).

Closely related Georgetown Salamanders ( *E. naufragia* ) and Salado Salamanders ( *E. chisholmensis* ) also demonstrate limited downstream distribution in small, headwater creeks in Williamson County, Texas (Pierce et al. 2010; Gutierrez et al. 2018). However, in the nearby Bull Creek watershed, *E. tonkawae* is not restricted to spring outlets and they occur in creek segments between and downstream of springs (Bendik et al. 2016). *Eurycea tonkawae* additionally utilize gaining creek segments that are not discrete springs and areas without noticeable groundwater input (Bendik et al. 2016; Adcock, MacLaren, et al. 2020). The Bull Creek watershed has most of the known‐occupied *E. tonkawae* sites (Figure 2; U.S. Fish and Wildlife Service 2013b), and its creeks are characterized by many springs, seeps, gaining segments, and areas of alluvial water storage and resurgence (Davis et al. 2001; Bendik et al. 2016; U.S. Fish and Wildlife Service 2021). The location of groundwater influence is likely to spatially vary depending on aquifer level, and Davis et al. (2001) reported that documenting the numerous sources of groundwater input in this system was infeasible, even though they expected the proximity to these sources influenced surface salamander abundance. The Bull Creek tributaries have extended groundwater influence along their lengths which not only helps regulate surface habitat conditions (Sweet 1982; Hubbs 1995; Power et al. 1999) but also provides additional migration corridors between the surface and subsurface habitat, which is important for these taxa (Bendik and Gluesenkamp 2013; Bendik et al. 2021). This appears to increase the area of appropriate habitat compared to the small, headwater creeks included in our study. We note that our study site with the furthest downstream detections, Avery Springhouse, was the only site where we noticed downstream groundwater input, albeit a minimal amount.

Within the Bull Creek system, Bendik et al. (2016) documented a higher proportion of juvenile *E. tonkawae* 80 m downstream of a spring outlet compared to adults, and they reported that it was unclear if this observation was related to juveniles avoiding adults, dispersal events, or larval drift. We did not observe any differences in the mean detection distance among salamander classes, but we also acknowledge small sample sizes for gravid females and juveniles.

We do not have data on local aquifer levels associated with our study sites, but aquifer levels are generally influenced by accumulated rainfall. According to the U.S. Drought Monitor (USDM), 37% of our study timeframe occurred during normal or wet conditions, 25% during abnormally dry conditions, and 38% during moderate to extreme drought conditions (U.S. Drought Monitor (USDM) 2025). We do not expect that our abundance and downstream distribution results are biased by aquifer conditions, as our study spans both normal and drought conditions.

### 4.2 Movement

We observed limited movement of *E. tonkawae* in our headwater creek study sites compared to the *E. tonkawae* movement rates observed by Bendik et al. (2016) in a Bull Creek tributary. Almost 90% of recaptured animals moved <5 m from their previous capture location and only 2.7% (*n* = 2) moved ≥19.5 m during our 36‐month study. In comparison, Bendik et al. (2016) reported 26% (*n* = 21) of recaptured *E. tonkawae* moved ≥20 m over just 4 months, and one individual moved 500 m over 4 years at Lanier Spring in Travis County.

Our observed movement patterns are more similar to those of *E. naufragia* and *E. chisholmensis* from similarly structured small, headwater creeks (Pierce et al. 2014; Gutierrez et al. 2018). Only 23.9% of *E. naufragia* and 17.5% of *E. chisholmensis* moved outside of their initial 5 m section over 32 months (Pierce et al. 2014; Gutierrez et al. 2018). Our study and Pierce et al. (2014) both document limited salamander movement despite large flood events occurring during the study timeframes. Low movement rates are documented for other salamanders in headwater systems including *E. bislineata* in Ohio (Ashton Jr. and Ashton 1978), *Pseudotriton ruber* in North Carolina (Cecala et al. 2009), and *Gyrinophilus porphyriticus* in New Hampshire (Lowe 2003, 2010; Cosentino et al. 2009).

We recaptured few salamanders outside of the surface CHU distance, but these recaptures appear to account for a disproportionate amount of salamander movement ≥5 m. We encourage future work to determine if *E. tonkawae* movement increases with distance from a spring outlet.

We suspect that habitat type (i.e., headwater creek versus gaining creek) influences downstream abundance and density patterns, downstream occurrence, and movement in *E. tonkawae* and closely related species. Similarly, other studies have also determined that habitat type influences salamander population parameters in eastern North America where interconnected first‐order headwater systems demonstrate higher salamander occupancy and abundance than first‐order systems that are isolated or flow directly into larger creeks and rivers (Lowe and Bolger 2002; Grant et al. 2009). We encourage future work that directly tests and compares central Texas *Eurycea* population parameters between headwater and gaining creeks.

### 4.3 Federally Designated CHU

All federally threatened and endangered species are required to have critical habitat designated, and CHUs are intended to protect areas that contain “physical and biological features essential to the conservation of the species” (U.S. Fish and Wildlife Service 2013b). It is prudent to recognize that species occurrence is not required for an area to have conservation value. For example, normally unoccupied areas maybe important for dispersal, prey base habitat, or other aspects of a species' life history. The designation of CHUs is an important part of the federal listing process, and it has lasting policy implications. Inadequate identification of critical habitat is often the result of limited data (Camaclang et al. 2015). We detected *E. tonkawae* outside of the 80 m surface CHU at three of the four sites that extended past this distance: Avery Springhouse, MacDonald Well, and PC 1 (Figures 3 and 4). At some of our study sites, it is possible that the currently unoccupied portions of the surface CHU provide conservation value or even may become occupied through natural changes to the habitat or habitat restoration efforts (e.g., Bendik et al. 2021). In contrast, at Hill Marsh, almost half of the surface CHU is a golf course pond that is uninhabitable and unlikely to have any conservation value for the species. Therefore, the uniform CHUs result in omission (i.e., failure to identify areas that may be critical) and may also result in commission (i.e., incorrect identification of non‐critical areas as critical habitat) errors (Camaclang 2015). Importantly, the downstream extent of *E. tonkawae* and the rate of change in relative abundance and relative density varied among sites, which is incongruent with the uniform federal surface critical habitat distance. These findings are important for other central Texas *Eurycea* salamanders, as the USFWS designated the same 80 m surface critical habitat boundaries for *E. naufragia* and *E. chisholmensis* (U.S. Fish and Wildlife Service 2021).

Central Texas *Eurycea* surface habitat has historically been considered “proximate to a spring outlet” (e.g., Sweet 1982; Chippindale and Price 2005). The combined results of this study, Bendik et al. (2016), and Adcock, MacLaren, et al. (2020) suggest this should more appropriately be considered “proximate to groundwater influence”. However, our relative abundance and relative density estimates demonstrate that “proximate” is site‐specific. Additional work is needed to determine the mechanisms that cause reduced abundance and limit the distribution of *E. tonkawae* downstream of groundwater influence which, accordingly, will better inform conservation decisions, management actions, and identification of surface critical habitat.

## Author Contributions

**Zachary C. Adcock:** conceptualization (lead), data curation (lead), formal analysis (lead), investigation (lead), methodology (lead), project administration (equal), writing – original draft (lead), writing – review and editing (lead). **Andrew R. MacLaren:** data curation (supporting), formal analysis (supporting), investigation (supporting), writing – original draft (supporting), writing – review and editing (supporting). **Michelle E. Adcock:** data curation (supporting), formal analysis (supporting), investigation (supporting), writing – original draft (supporting), writing – review and editing (supporting). **Michael R. J. Forstner:** conceptualization (supporting), funding acquisition (lead), project administration (equal), writing – original draft (supporting), writing – review and editing (supporting).

## Conflicts of Interest

The authors declare no conflicts of interest.

## Data Availability

All data and code associated with this study are available from Dryad: https://doi.org/10.5061/dryad.n5tb2rc5b.
